# Evolving culturally competent veterinary care: a community-based partnership with the Santee Nation

**DOI:** 10.3389/fvets.2025.1652546

**Published:** 2026-01-07

**Authors:** Ronald J. Orchard, Deon LaPointe, Sara Miner, Cayley Conrad, Chris Blevins, Emmanuel Jeje

**Affiliations:** 1 Department of Clinical Sciences, College of Veterinary Medicine, Kansas State University, Manhattan, KS, United States; 2 Santee Nation, Niobrara, NE, United States; 3 Staley School of Leadership Studies, Kansas State University, Manhattan, KS, United States

**Keywords:** cultural competence, One Health, community-based partnership, veterinary outreach, tribal health, decolonizing education, land-grant institutions, service-learning

## Abstract

Access to veterinary care remains a profound equity issue across the United States, particularly in Indigenous communities where animals hold vital cultural and spiritual significance. This case study examines a longitudinal, community-based partnership between Kansas State University’s College of Veterinary Medicine and the Santee Nation, offering a model of culturally competent veterinary outreach grounded in the principles of One Health, cultural humility, and decolonizing education. Over 7 years, the program has evolved from a small animal service-learning initiative into a multifaceted, community-guided collaboration that integrates student learning, tribal leadership, and interprofessional engagement. Key programmatic components include structured cultural preparation, provision of mobile veterinary care—including equine services prioritized by the community—relationship-centered practices, and ongoing feedback loops. Students engage in implicit bias assessments, autoethnographic reflection, and historical learning about land-grant institutions and tribal sovereignty. Clinical interactions emphasize relational accountability, transparency, and the centering of Indigenous knowledge. Community members, in turn, act as co-educators and co-designers, reshaping what competent veterinary care looks like within a tribal context. Findings illustrate how veterinary outreach, when framed through reciprocal partnership rather than charity, can build trust, improve access, and foster professional identity formation rooted in equity. This model aligns with national calls for culturally responsive care and offers a replicable framework for institutions seeking to reimagine their role in tribal health and veterinary education. The program advances the scholarship of engagement by demonstrating how veterinary institutions can co-create just and sustainable systems of care alongside historically excluded communities.

## Introduction

Access to veterinary care in the United States is increasingly understood as an issue not only of animal welfare, but of public health and social justice. Over 20 million pets live in households experiencing poverty, and more than 70% of these animals have never received veterinary care (1). These disparities result in untreated illness, suffering for animals, emotional hardship for pet guardians, and broader community-level impacts—especially among communities already facing systemic exclusion.

A growing body of research has documented the spatial and structural dimensions of veterinary access inequities. Neal and Greenberg’s Veterinary Care Accessibility Score (2) highlights entire regions—including the rural Midwest, Southwest, and tribal lands—as “veterinary deserts,” where geography, cost, and social marginalization converge. Reese and Li (3) similarly describe “animal welfare deserts,” drawing parallels to food and healthcare inequality. Together, these findings underscore that veterinary care inaccessibility is not random, but the result of longstanding disinvestment and racialized policy decisions.

For Indigenous communities, the consequences of this inequity are particularly acute. Among the Santee Dakota, as in many tribal nations, animals—especially horses and dogs—hold profound emotional, cultural, and kinship significance (4, 5). Veterinary care in these contexts is not simply clinical; it is relational and spiritual. When companion animals suffer or die from preventable conditions, the trauma reverberates through families and across generations. Mainstream veterinary models, grounded in Western biomedical assumptions, often fail to accommodate these deeper relationships or respond to culturally embedded understandings of health (6, 7).

These challenges are compounded by the enduring structural effects of settler colonialism. Land-grant universities—while funded by the expropriation of Indigenous lands—have historically failed to extend educational and healthcare equity to tribal communities (8, 9). While veterinary schools may offer outreach services, they are often unidirectional, short-term, and transactional. As Orchard et al. (10) note, these approaches can reflect ethical misalignments—rooted in institutional authority, rather than reciprocal partnership.

Addressing these layered inequities requires more than increased service provision. It demands models of engagement grounded in cultural humility, Indigenous sovereignty, and justice-oriented pedagogy. National research has increasingly emphasized this need. For example, the Delphi study on the “Top 20 Questions to Advance Access to Veterinary Care” identified culturally relevant, community-defined interventions as a central priority (11). This case study responds directly to that call.

We describe a 7-year community-campus partnership between Kansas State University’s College of Veterinary Medicine and the Santee Nation. What began as a small animal outreach effort has evolved into a culturally grounded, One Health–informed, interprofessional program shaped by tribal leadership and co-designed to meet community needs. Rather than positioning the university as the primary source of expertise, the program embraces decolonizing educational frameworks that redistribute interpretive authority (12, 13). Indigenous leadership defines care priorities, informs pedagogical design, and directs evaluation practices.

This reframing of outreach aligns with what Orchard et al. (10) call “partnership choreography,” where institutions and communities move together—sometimes uneasily—toward equity. It also exemplifies restorative community praxis, as articulated by Asadullah (14), by recognizing veterinary care not only as service, but as an act of healing, reparation, and cultural continuity.

The program reflects One Health principles that emphasize the interdependence of human, animal, and environmental health. Yet it also pushes One Health beyond its conventional biomedical parameters by integrating Indigenous epistemologies and affirming that animals are relatives, not merely patients (15). Through interprofessional collaboration with social workers and community health practitioners, the program addresses social determinants of health—including housing insecurity, transportation access, and trauma.

Educationally, the program fosters reflective practice through guided journaling, cultural context readings, and immersive service-learning. For students—particularly those unfamiliar with tribal histories or structural inequity—this work can be transformative. One Indigenous student’s reflection described veterinary medicine not as a neutral profession, but as a pathway to communal healing and cultural reclamation. These experiences underscore the potential of veterinary education to cultivate equity-oriented practitioners.

This manuscript offers a detailed case study of the Santee partnership: its development, pedagogical strategies, community collaborations, and lessons learned. We aim to contribute to a broader conversation about how veterinary schools can move from extractive outreach models to reciprocal, culturally responsive systems of care—co-produced with the communities they serve.

### Author positionality statement

As authors, we acknowledge our diverse social locations, professional roles, and institutional affiliations that shape how we engage in this work. We write from within veterinary academia, based at a land-grant institution, and are predominantly non-Indigenous. Our access to Indigenous communities, including the Santee Nation, is made possible through trust built over years of partnership—not entitlement. We recognize that our positionality affords us privileges within academic and professional systems, and we aim to use this position to elevate community voice, center reciprocal learning, and challenge extractive or charity-based paradigms of service.

This manuscript reflects an evolving effort to enact cultural humility—not as a fixed skill but as a continuous practice of self-reflection, power analysis, and relationship accountability. We acknowledge that we are not neutral observers; our perspectives are shaped by our training, our institutional imperatives, and our histories. We aim to engage in this work responsibly, listening first, deferring where appropriate, and being transparent about the limitations of our perspective.

### Land-grant acknowledgement

Kansas State University, where this program is based, is a land-grant institution established through the 1862 Morrill Act. The lands used to fund the university were expropriated from Indigenous Nations, including through processes of coercion and violence. This foundational history is inseparable from our current work. The irony—and responsibility—of conducting veterinary outreach with Indigenous communities under a land-grant banner is not lost on us.

We acknowledge the Santee Nation and the broader Dakota peoples whose lands and sovereignty have been disrupted by federal policies and settler colonialism, including those that enabled the rise of land-grant universities. We offer this acknowledgment not as a symbolic gesture but as a call to accountability. We commit to using our institutional platforms to support Indigenous self-determination, expand access to culturally competent care, and work toward restorative justice within our educational systems.

### Context

The Santee Sioux Nation, located in northeastern Nebraska, is home to approximately 927 residents across 450 square kilometers. The reservation’s geography and variable climate—hot summers, cold winters, and increasing unpredictability due to climate change—compound long-standing infrastructural vulnerabilities. These conditions intersect with a deep historical legacy of displacement, trauma, and resilience.

Originally from present-day Minnesota, the Santee were forcibly relocated to Nebraska in 1866 following the 1862 U.S.-Dakota War and the subsequent mass execution of 38 Santee men—the largest in U.S. history. Successive displacements, unjust treaties, and environmental degradation at relocation sites contributed to profound losses. Yet despite this legacy, the Nation has established a strong foundation of self-governance, exemplified by the development of a multispecialty health center and an Office of Environmental Protection, which integrates traditional knowledge and climate adaptation planning.

Veterinary care remains difficult to access. The nearest full-service clinics are over 60 miles away, and limited transportation options, high costs, and systemic underinvestment exacerbate the challenge. The cultural significance of animals—especially dogs and horses—intensifies the stakes. Within the Santee Nation, animals are not merely pets; they are kin, protectors, and ceremonial beings, central to emotional, educational, and spiritual life. Thus, inaccessible veterinary care represents more than a service gap—it is a threat to cultural and communal well-being.

This community-based veterinary program brings together stakeholders across several groups. Participants include:

- Veterinary students from Kansas State University (KSU), primarily in their clinical training year, who participate through an elective service-learning course;
- Veterinary faculty and clinical instructors who supervise care, provide context, and facilitate student learning;
- Santee community leaders, including tribal council members, cultural educators, and health officials, who co-design and guide the program;
- Pet guardians and residents, who voluntarily participate in clinics and share their perspectives on compassionate care;

Prior to their visit, students engage in structured cultural preparation, including readings on tribal sovereignty, the history of land-grant institutions, and ethical outreach practices in Indigenous contexts. These preparatory materials foster cultural humility and encourage students to understand veterinary care not as transactional, but relational.

On-site, students deliver basic veterinary services under supervision, engage in conversations with pet guardians, and reflect on the broader social determinants of animal and human health. Community members frequently describe how inaccessible care has forced reliance on home remedies or peer advice—but also express strong interest in preventative care and health education when delivered with respect and continuity.

Events are held at accessible community hubs, such as the health center. The program’s continuity over multiple years has cultivated trust and allowed for co-evolution with community priorities—including the integration of equine care. Tribal leaders guide program focus and content, reinforcing the primacy of sovereignty, relationship, and cultural continuity over charity-based models.

### Key programmatic elements

The community-based veterinary partnership between Kansas State University’s College of Veterinary Medicine (KSU-CVM) and the Santee Nation operates through a multifaceted structure rooted in cultural responsiveness, co-governance, and iterative learning. Over several years, the program has evolved through feedback, reflection, and adaptation, leading to five critical programmatic elements: (1) cultural preparation and reflection, (2) clinical care delivery, (3) community engagement and trust-building, (4) infrastructure and coordination, and (5) formative feedback loops. These elements are not neutral technical features but emerge from a larger framework of relational practice and power redistribution, as articulated in Orchard et al. (10) vision of “dancing toward equity” in land-grant engagement.

#### Cultural preparation and reflection

Student preparation prior to site visits is central to the program’s integrity and impact. In alignment with Tervalon and Murray-García’s (16) foundational model of cultural humility, the program emphasizes reflection and structural awareness over checklist-style competency acquisition. Preparation is approached as a continuous, ethical practice grounded in relationships, not technical mastery.

Students complete assigned readings that detail the historical and political context of the Santee Sioux Nation, including settler-colonial violence, land dispossession, and the legacy of broken treaties. These are complemented by implicit bias testing and guided post-test discussions that challenge students to examine their assumptions about professionalism, neutrality, and authority.

To deepen this work, the program incorporates modules adapted from the University of Oregon Cultural Humility Toolkit (17). These include identity mapping, power analysis, and narrative reflection exercises that frame veterinary care as embedded in colonial and institutional histories. Unlike conventional cross-cultural trainings, the toolkit promotes community-defined notions of wellness and accountability.

This approach reflects Orchard et al.’s (10) argument that cultural humility must “redistribute interpretive authority” and foster student engagement with Indigenous worldviews. Preparation, in this model, is not peripheral—it is the ethical entry point to community-based veterinary care.

#### Clinical care delivery

Veterinary care is provided through multi-day mobile clinics hosted at the Santee Nation’s multispecialty health center. Services include vaccinations, deworming, wellness exams, minor treatments, and owner education. These visits are designed not as episodic outreach but as part of an ongoing relational partnership, with students returning year over year under the supervision of consistent faculty leads.

Students participating in this component are primarily in the clinical stage of their education, typically in their final year, and enroll through elective rotations in shelter medicine and community outreach. They are responsible for patient intake, medical assessments, and communication with pet guardians, all under faculty supervision. Faculty model low-barrier, client-centered communication, and students are coached in adapting technical language to ensure clarity, respect, and shared understanding.

In response to community feedback, the program has expanded to include equine services. Horses are of spiritual, cultural, and economic significance to the Santee people, and their inclusion required logistical adaptations in safety, equipment, and team composition. This expansion underscores the program’s commitment to community-defined priorities and non-extractive clinical practice.

Rather than privileging volume or procedural speed, clinical care is guided by an ethic of presence, flexibility, and informed consent. Students are encouraged to view veterinary medicine as a relational encounter, shaped by trust and context as much as by diagnosis and treatment. This approach affirms the core principle that care must be both clinically sound and culturally attuned.

#### Community engagement and trust-building

The program’s success is grounded in a commitment to sustained, reciprocal relationships with the Santee Sioux Nation. Rather than treating community participation as an accessory to clinical service, the partnership centers tribal sovereignty and local expertise across all phases of engagement. Tribal leadership plays a co-governance role in setting priorities, coordinating logistics, and advising on educational design. This approach aligns with models of culturally humble leadership that emphasize shared authority, responsiveness, and sustained relationship-building (18).

Community members are active agents in shaping the veterinary learning environment. Pet guardians often engage in reciprocal teaching, sharing care practices, oral histories, and local environmental knowledge. These exchanges challenge the traditional unidirectional flow of expertise in outreach and instead affirm community-based knowledge as central to both care and curriculum. In this context, veterinary knowledge becomes dialogic, not prescriptive, and students are invited to see clients not as passive recipients but as collaborators in the co-production of care.

Consistency in faculty leadership and returning student volunteers deepens this relational infrastructure. Many community members greet team members by name, recall prior visits, and share updates on their animals. These longitudinal interactions foster mutual recognition and a sense of shared investment in health outcomes. Such relational continuity is rare in short-term outreach programs and reflects a long-haul orientation to engagement.

While formal community advisory structures have not yet been implemented, informal feedback mechanisms operate dynamically. On-site conversations, observations, and post-event discussions allow for iterative refinement of services and student preparation. These practices reflect what Orchard et al. (10) describe as the “re-narration” of partnership roles, wherein Indigenous communities articulate expectations, define relevance, and guide the focus of institutional labor.

Ultimately, trust-building in this program is not a soft outcome but a structural commitment. As Haraway might suggest, staying with the trouble means choosing entanglement over distance and co-responsibility over neutrality (19). In this model, engagement is not a singular event but an ongoing ethical relationship sustained through reciprocity, humility, and deep listening.

#### Infrastructure and coordination

Successful mobile veterinary service delivery requires a robust infrastructure that is both materially efficient and ethically aligned with community-centered principles. Each trip to the Santee Sioux Nation involves the careful orchestration of transportation logistics, equipment preparation, clinical material management, and schedule coordination. This work is not ancillary—it reflects a deeper ethic of relational and logistical accountability.

Faculty and students work together to transport examination tables, sharps containers, vaccination supplies, and waste management equipment, often loading these into a retrofitted van or trailer. Cold chain protocols are maintained for biological products such as vaccines, and power sources are secured in advance to support diagnostics or surgical tools. Roles are pre-assigned to students to facilitate both efficiency and experiential learning. These operational practices are not merely procedural—they socialize students into the labor and planning that undergird equitable care delivery.

The partnership benefits significantly from the use of the Santee Nation’s health center, which offers a climate-controlled, culturally resonant, and centrally located space for care. The site includes access to restrooms, running water, electricity, and tribal staff who assist with coordination. This shared use of space reaffirms the Nation’s role not as a beneficiary, but as a host and co-director of the program. Such attention to local infrastructure counters the colonial tendency of external resource extraction and reinforces mutual investment (10).

Scheduling is another area where coordination reflects cultural responsiveness. Visits are planned around community calendars to avoid conflicts with ceremonies, school events, or harvest periods. In recent years, extreme weather and road conditions have also been factored into planning, with seasonal shifts and climate concerns requiring increasing flexibility. These logistics embody what others have called “contextual equity”—the alignment of service delivery with the lived rhythms of a particular place (20).

Even seemingly technical decisions—such as where to set up an exam table or how to dispose of medical waste—are approached through a lens of care. As Orchard et al. (10) argue, logistics are political: they reveal whose labor is prioritized, whose time is respected, and whose expertise is operationalized in the design of outreach programs.

By embedding infrastructure within a framework of partnership and responsiveness, the program shifts the narrative away from one of delivery and toward one of mutual stewardship. Coordination is thus both a material and ethical act—linking the physical conditions of care to the values that animate community engagement.

#### Formative feedback loops

Evaluation within the KSU–Santee Nation partnership is not an end-point exercise of summative judgment; rather, it is conceptualized as an iterative, formative, and dialogic process that sustains the relational fabric of the program. Feedback loops are intentionally structured to amplify reflection, reciprocity, and shared learning among students, faculty, and community members.

At the conclusion of each service trip, students and faculty engage in a facilitated debriefing process. These sessions move beyond clinical outcomes to explore cultural, emotional, and ethical dimensions of the experience. Participants are encouraged to reflect critically on their positionality, assumptions, communication practices, and the structural factors shaping the delivery of care. These reflections often surface tensions embedded in veterinary education—such as the prioritization of efficiency over presence, or expertise over humility—and allow students to reframe their emerging professional identities in light of community-defined values (10).

In addition to faculty-led debriefs, students submit written reflections that are reviewed by program instructors. These narratives serve both pedagogical and programmatic functions, offering insight into how students are metabolizing the experience and highlighting areas for curricular adaptation. For example, student writing has contributed to recent changes in the pre-trip curriculum, such as the inclusion of trauma-informed care principles and more explicit discussion of land-grant histories.

Although the program currently relies on informal mechanisms—such as verbal feedback from pet guardians and real-time observations by tribal partners—it is moving toward more systematic approaches. Beginning in 2025, the team plans to incorporate structured evaluative tools, including anonymous community surveys and qualitative interviews with tribal liaisons and veterinary students. These instruments are being developed collaboratively to ensure that the measures reflect community priorities and avoid extractive evaluation practices.

Crucially, feedback is not understood as a unidirectional flow of information. Rather, it functions as what Orchard et al. (10) describe as “a mode of being in partnership”—an ongoing process of attunement, renegotiation, and co-design. By treating feedback as a relational practice rather than a metric, the program resists the bureaucratization of evaluation and instead reclaims it as a space for ethical deliberation and mutual transformation.

In this way, the formative feedback loops operationalize the decolonizing ethos of the program. They sustain a culture of responsiveness that is both humble and rigorous, enabling the partnership to evolve not only in content but in its very approach to care, learning, and accountability.

## Discussion

This community case study illustrates a culturally engaged model of veterinary outreach that emphasizes reciprocal learning, community-defined priorities, and curricular integration of cultural humility. These features position the program not only as a service delivery intervention but as a scalable educational innovation aligned with evolving mandates in health professions education to address disparities and promote cultural competence.

### A community-engaged model of veterinary outreach

This community case study illustrates a culturally engaged model of veterinary outreach that emphasizes reciprocal learning, community-defined priorities, and the curricular integration of cultural humility. These features position the program not merely as a service delivery intervention but as an educational innovation aligned with evolving mandates in health professions education to address health disparities and foster relational care.

### Reframing veterinary engagement through cultural competency

Veterinary outreach programs are often evaluated by clinical metrics, yet the persistent gaps in access—particularly in Indigenous communities—demand an expanded lens of cultural competence. Defined as the ability of health professionals to communicate with and effectively provide care to patients from diverse sociocultural backgrounds, cultural competence has gained widespread support across the health professions as a pathway to mitigating disparities (19).

In veterinary medicine, this imperative is echoed by the AVMA, which acknowledges that sociocultural barriers impede care access and that cultural competency is a realistic, short-term strategy to improve outcomes for underserved populations (21). Our program responds to this call by embedding cultural humility across all phases of engagement—through required readings on Indigenous history, self-assessment tools like the Cultural Humility Toolkit (17), structured reflection exercises, and feedback sessions co-led by community members.

These curricular elements align with Bhui et al.’s (20) emphasis on interventions that do not merely raise awareness but reconfigure institutional norms. By prioritizing relationship-building, contextual learning, and student reflection, the program shifts the emphasis from content delivery to identity formation, fostering what Tervalon and Murray-García describe as a “lifelong commitment to self-evaluation and critique” (16).

### Cultural competency as system-level transformation

What emerges from both the Like (19) and Bhui et al. (20) reviews is that cultural competence is most impactful when applied not as a standalone training but as a system-wide shift. This case study supports that conclusion. Through its sustained partnership with the Santee Nation and its emphasis on student reflection, faculty commitment, and institutional support, the program contributes to a broader culture of equity within veterinary education.

As described in Orchard et al. (10), the most profound learning happens not when content is delivered but when institutions learn to “step back” and let community-defined priorities shape the choreography of engagement. This approach requires faculty and students to recognize the asymmetries of power embedded in land-grant outreach and to move beyond the logic of service provision toward a more relational, justice-oriented practice. It reframes veterinary engagement as an evolving partnership—one where knowledge is shared, responsibilities are negotiated, and community leadership is centered.

Students do not only learn about cultural humility—they witness it in action as tribal leaders host, guide, and inform the care delivery process. This relational pedagogy challenges students to recognize the limits of their knowledge and invites them into a mode of learning grounded in respect, humility, and shared authority—principles identified as central to effective cultural competency training (16, 19).

### Sustaining competency through institutional infrastructure

Cultural competence, to be effective, must move beyond one-time workshops and become structurally embedded. As Like (19) notes, piecemeal efforts often falter due to inconsistent implementation, lack of outcome tracking, and minimal faculty development. In contrast, our program situates cultural engagement within a repeatable, experiential, and multi-stakeholder framework that incorporates student coaching, cross-sectoral dialog, and longitudinal partnerships.

The program’s alignment with the U.S. Office of Minority Health’s CLAS standards (22)—particularly regarding culturally appropriate services, community governance, and equity evaluation—offers a replicable structure. In this context, cultural competency is not simply an educational objective but a system-level value, one that undergirds decisions about whom we serve, how we define learning, and which forms of knowledge we center.

Comparatively, the University of Sydney’s culturally responsive veterinary curriculum similarly integrates community ethics, belief systems, and lived experience as core content alongside clinical training (23). Our model parallels these efforts by treating cultural humility as foundational—not ancillary—to effective veterinary practice.

### Cultural humility as relational practice

At the heart of our program is the commitment to relational pedagogy. Students do not merely learn about cultural humility; they experience it in practice through interactions with tribal elders, community liaisons, and clients whose insights guide the delivery of care. This dynamic, described by Orchard et al. (10), requires institutions to “step back”—allowing community-defined priorities to reshape not only services but educational trajectories.

This model challenges the traditional land-grant logic of top-down service delivery and instead promotes a dialogical, justice-oriented model of care. In doing so, students come to recognize the limits of their clinical training and the importance of humility, trust, and cultural fluency. As echoed in Tervalon and Murray-García (16), these are not checkboxes but evolving commitments that call for ongoing interrogation of power, privilege, and responsibility.

### Limitations and challenges

Despite its promise, the program faces limitations. First, it is currently restricted to students in their clinical year, limiting longitudinal exposure across the veterinary curriculum. Second, implementation depends heavily on faculty with community engagement expertise and partnerships that are time-intensive to build and sustain. Third, community feedback mechanisms, while present, require further formalization to ensure bidirectional accountability and continuous improvement. Lastly, evaluating cultural humility and relational competencies remains methodologically complex, and further mixed-methods research is needed to capture learning outcomes across affective and behavioral domains.

### Implications and recommendations for veterinary curricula

Veterinary schools seeking to integrate cultural competence should consider several key design principles drawn from this case:

1. *Embed cultural humility early and longitudinally*—moving from isolated trainings to scaffolded, developmental models across the curriculum.
2. *Center community voices as co-educators*—by establishing sustained partnerships and incorporating feedback from clients, tribal leaders, and community health workers.
3. *Invest in faculty development*—to ensure that reflective dialog, facilitation, and trauma-informed teaching are supported.
4. *Align curricular efforts with national standards*—such as the CLAS framework and CBVE 2.0 competencies that address communication, collaboration, and diversity.

Adopting these approaches requires more than curricular modification; it necessitates a cultural shift within institutions. As health professions education increasingly emphasizes equity and inclusion, veterinary medicine must follow suit—not only to serve communities more ethically but to prepare graduates for the complexities of real-world practice.

### Final reflections

The Santee Nation case underscores that culturally competent veterinary outreach is not a supplemental enrichment—it is a professional imperative grounded in ethics, effectiveness, and equity. Programs like this demonstrate that service-learning can move beyond skill-building into the realm of transformative education. By centering humility, accountability, and relationship, veterinary schools can align their land-grant missions with the deeper call to solidarity and justice.

## Data Availability

The original contributions presented in the study are included in the article/supplementary material, further inquiries can be directed to the corresponding author.
